# Local and systemic humoral immune responses to *Histophilus somni* recombinant antigens administered intranasally and subcutaneously to dairy calves

**DOI:** 10.1038/s41598-024-78605-x

**Published:** 2024-11-11

**Authors:** Joanna Bazjert, Paulina Jawor, Maciej Pisarek, Rafał Baran, Wojciech Jachymek, Tadeusz Stefaniak

**Affiliations:** 1 Department of Immunology, Pathophysiology and Veterinary Preventive Medicine, Wrocław University of Environmental and Life Sciences, C.K. Norwida 31 Str, Wrocław, 50-375 Poland; 2 Hirszfeld Institute of Immunology and Experimental Therapy, Polish Academy of Science, Rudolfa Weigla 12 Str, Wrocław, 53-114 Poland

**Keywords:** rHsp60 *H. somni*, rOMP40 *H. somni*, CpG ODN2007, MPLA, Immunology, Microbiology, Diseases

## Abstract

Bovine respiratory disease (BRD) causes significant economic losses in dairy calves. Induction of an early immune response via parenteral vaccination is complicated by the interference of colostral immunity. In this study, we investigated early immunization against selected conserved bacterial antigens. Calves were vaccinated twice intranasally and then subcutaneously with *Histophilus somni* recombinant proteins (rOMP40 or rHsp60) mixed with one of two adjuvants: CpG ODN2007 or MPLA. The control group (Con) was treated with PBS. The first immunization was done between 24 and 48 h of life and then twice in two weeks intervals. Blood, nasal, and saliva secretion samples were collected directly before vaccination (S1–S3) and then on 42–44 (S4) and 59–61 (S5) day of life. Antibodies (IgG_1_/IgG_2_/IgM/IgA in serum; IgG_1_/IgA in secretions) against both vaccine antigens were quantified in all samples. Intranasal and subcutaneous vaccinations using the described formulas did not increase antibody reactivity against the tested proteins. The reactivity of serum IgG_1_, IgM, and IgA anti-rOMP40 antibodies was significantly higher in S1 in all groups than that in the other samplings (p˂0.01). Significant differences in the reactivity of serum anti-rOMP40 antibodies between groups were identified in S1 (IgA reactivity was higher in the CpG vs. MPLA group; *p* < 0.05), S4 (IgM reactivity was higher in Con vs. CpG group; *p* < 0.05), and S5 (IgG1 reactivity was higher in MPLA vs. Con group; *p* < 0.05). The lack of consistent changes in antibodies after immunization (S4 and S5) hinders the drawing of conclusions regarding the effect of immunization on antibody reactivity. In the future, establishing a proper immunization window and adjuvants for nasal vaccines against bacterial pathogens causing BRD in calves remains to be determined.

**Supplementary Information:**

The online version contains supplementary material available at 10.1038/s41598-024-78605-x.

## Introduction

Mucosa-associated lymphoid tissue (MALT) is the first immune defense barrier against more than 90% of potential pathogens, and represents the largest immune organ in the body^1^. The MALT in the bovine upper respiratory tract (URT) includes nasal-associated lymphoid tissue (NALT) and lymphoid tissues of Waldeyer’s ring, which include the lingual, palatine, pharyngeal, and tubal tonsils^1,2^.

NALT consists of T and B lymphocytes, dendritic cells, macrophages, and microfold cells located in the nasal cavity. In contrast to other mucosal tissues, the NALT develops after birth^3^. Its composition is established by an interaction with indigenous bacteria in the nasal cavity, which takes place from the first day of life^4^. Until now, the relationship between cells present in the inductive and effector sites of NALT has not been fully described^5^. However, NALT remains the first immune tissue to be exposed to inhalant antigens and pathogens, including bacteria, that cause respiratory diseases^3^.

Calf respiratory disease causes significant economic losses in the feedlot industry due to decreased production and increased costs associated with treatment^6–9^. On the other hand, subcutaneous/intramuscular immunisation of calves in the first weeks of life has been shown to be ineffective due to interference of maternal antibodies with vaccine antigens^10^. Therefore, the induction of early life immunity against bovine respiratory disease (BRD) pathogens (both viruses and bacteria) in the NALT seems to be one of the possible ways to prevent BRD in newborn calves. The intranasal route of immunization with antigens isolated from BRD-associated pathogens, appears to be attractive for several reasons:

- the high concentration of lymphoid tissue in the mature NALT structures^1,11^,
- the induction of interferon production and its secretion into nasal secretions^12^,
- the induction of the production of sIgA and sIgG antibodies against BRD pathogens^13,14^,
- possibility of inducing a local immune response against modified live virus and avirulent (whole bacterial) live culture despite the presence of maternal antibodies in calf serum^15–18^.

Several factors, including antigens, adjuvants, formulations, administration routes, and animal models, should be considered for the efficacy and safety evaluation of the immunization of mucous membranes^19^.

Cattle are vaccinated against BRD pathogens such as Bovine Herpesvirus-1 (BHV-1), Bovine Respiratory Syncytial Virus (BRSV), Bovine Parainfluenza-3 *virus* (PI-3 V), *Mannheimia haemolytica*, and *H. somni*, with combinations of modified live viruses, killed viruses, bacterins, or bacterial culture extracts^7^. However, only a few vaccines (mostly antiviral vaccines) can be effectively administered intranasally during the first week of life^2^. Vaccination against viruses alone is not effective in preventing this disease^7^. Therefore, it is necessary to search for new intranasal immunization formulas including bacterial antigens and suitable adjuvants/immunostimulants.

In the veterinary market, only one commercially available intranasal BRD vaccine provides protection against three viruses (BHV-1, BRSV, and PI-3 V) and two bacteria (*Pasteurella multocida* and *M. haemolytica)*. The Bovilis Nasalgen 3-PMH (Merck Animal Health) vaccine has been shown to be effective for intranasal vaccination of healthy cattle at one week of age or older and has demonstrated protective efficacy against *P. multocida* and *M. haemolytica *challenge. This vaccine is based on a modified live virus and avirulent (whole-bacterial) live culture^18^. An interesting alternative to intranasal immunization is the use of recombinant proteins instead of whole bacterial cells. This approach guarantees the selection of immunodominant antigens or cocktails of selected antigens purified from the pathogens^20^, the ability to induce antigen-specific antibody responses, the possibility of chemical modification, and low toxicity^19^. Therefore, in this study, two recombinant proteins from the BRD pathogen *H. somni* (outer membrane protein 40 kDa and heat shock protein 60 kDa) were used as antigens for the intranasal immunization of calves.

The bacterial outer membrane (OM) contains a variety of proteins^21^, some of which have conserved structures^22^. *H. somni *OM is composed of a wide range of proteins, one of them is OMP40 kDa (OMP40)^23^. OMP40 is immunogenic in rabbits^24 ^and calves^25^. The rabbit antiserum showed cross-reactivity with homologous proteins from different *H. somni *strains^24^. Calve *H. somni* anti-OMP40 antibodies revealed reactivity with homologous proteins expressed by *P. multocida*, *Escherichia coli* and *Salmonella *Enteritidis^25^.

Heat Shock Proteins (HSPs) are highly conserved proteins common in nature that have been identified in *Procaryota* and *Eucaryota*^26,27^. Hsp60 is continuously expressed on the surface of bacterial cells^27^. Hsp60 also plays an important role in *H. somni *biofilm formation^28^. To date, an extensive humoral immune response to Hsp60, originating from different gram-negative bacteria^29–31^ including *H. somni* and *P. multocida*^32^has been observed. Antibacterial Hsp60 antibodies have also been shown to demonstrate strong cross-reactivity^32,33^.

One of the most important elements that might have an essential impact on the nature of the immune response following administration of subunit protein-based antigens is the adjuvant/immunostimulant used in the immunization procedure^19^. Activation of the innate immune response by PAMPs (pathogen-associated molecular patterns) enhances the development of an acquired immune response against invading or invasive pathogens. Recent studies on the innate immune system have shown that PAMPs can be used as vaccine adjuvants in veterinary medicine^3,34–38^. CpG ODNs are synthetic oligodeoxynucleotides containing unmethylated CpG dinucleotides in particular sequence contexts (CpG motifs)^39^that are recognized by Toll-like receptor 9 (TLR9), leading to strong immunostimulatory effects^40^. Numerous investigators have shown that immunization via mucosal routes using CpG-ODNs induces mucosal and systemic immune responses to various antigens in cattle^37,41^. ODN 2007 is a class B CpG ODN with a preference for bovine and porcine TLR9^42^. CpG-ODN 2007 has strong immunomodulatory and immunostimulatory activities in sheep, goats, dogs, poultry, and a broad variety of important livestock and companion animals^41^. It strongly activates B cells but weakly stimulates IFN-α secretion^43^. Monophosphoryl lipid A (MPLA) is a low-toxicity component of LPS that retains the immunologically active lipid A portion of its parent molecule^44^. It is a PAMP that can induce both humoral and cellular immune responses after systemic and mucosal immunization^45^. MPLA is a potent activator of TLR4, with negligible TLR2 activity^46^. It has also been used in licensed human vaccines. Studies on MPLA as an adjuvant for commercial animal vaccines have yet to be reported^44^, but its usefulness as an immunostimulator during immunization in calves has been shown^38^.

We hypothesized that vaccine formulations involving *H. somni* rHsp60 and rOMP40 antigens mixed with one of the two adjuvants, CpG and MPLA, could induce a specific immune response against recombinant proteins in one or two day old calves despite potential maternally derived antibody interference. This study aimed to evaluate the local and systemic humoral immune responses to early vaccination of the nasopharyngeal mucosa, followed by subcutaneous immunization with selected conservative recombinant bacterial antigens mixed with CpG or MPLA adjuvants in dairy calves.

## Materials and methods

### Animals

This study was conducted on a commercial dairy farm with 240 Holstein–Friesian cows with a milk yield of 8600 kg per lactation. The cows in the herd were not vaccinated using commercially available vaccines against viral pathogens, such as Bovine Viral Diarrhea Virus (BVDV), BRSV, or PI-3. Against BHV-1 last herd vaccination was performed two years before the study. Thirty-three heifer calves, either Holstein-Friesian (*n* = 32, 11 in the control group) or Holstein-Friesian-Simmental crossbreeds (*n* = 1, one in experimental group II), were enrolled in the study. There were 11 calves in each group (experimental group I, CpG; experimental group II, MPLA; control, Con). Successively born calves were enrolled to individual groups (in the period of January 2020–September 2020).

Each calf was fed on the first day with dam colostrum within 6 h of life (2–4 L at first feeding; colostrum samples were collected by the farm staff and stored at – 20 °C), then up to five days of life with transition milk, and from day 6 were fed with a milk replacer (ASCOMILK F SUPER TOP, Noack Group GmbH; Wien, Austria) twice daily with 3 L until approximately ~ 59–61 days of age (the end of the experiment). For the first 14 days, calves were kept in individual pens and moved to group pens (six in each). The calves in the pen group had ad libitum access to pellets (30% crude protein, 7.4% fiber, 5% fat, 8.79% ash, and 5.5% carbohydrates) mixed with whole grains of oat and corn in a ratio of 2:1:1. The animals were under permanent care of a farm veterinarian. Thirteen calves between 5 and 9 weeks of age were disbudded by cauterization with hot iron (see repository files).

### Immunization and sampling

Calves were vaccinated with recombinant proteins from *H. somni* (rHsp60 and rOMP40) mixed with one of two adjuvants: CpG ODN 2007—Class B CpG oligonucleotide bovine/porcine TLR9 ligand or MPLA-Monophosphoryl lipid A from *Hafnia alvei* strain 1200 (Polish Collection of Microorganisms; PMC), with structures identical to *Escherichia coli *MPLA. CpG ODN 2007 was synthetized by Oligo.pl (Warsaw, Poland), and MPLA was obtained as previously described^47^. Recombinant proteins were produced by Pure Biologics S.A. [Wrocław, Poland]. The protein production process has been described by Bajzert et al.^25,32^. Two compositions were used for immunization.

1. rOMP40 + rHsp60 + CpG ODN2007 - experimental group I = CpG, *n* = 11;
2. rOMP40 + rHSP60 + MPLA - experimental group II = MPLA, *n* = 11.

The vaccine composition, dose, route of administration, and treatment groups are summarized in Table 1. All components were dissolved in phosphate-buffered saline (PBS) pH = 7.4 (Fisher BioReagents; Bruxelles, Belgium), prepared using endotoxin-free ultrapure water (EMD Millipore Corp., Germany). The control group was treated with PBS alone (Con, *n* = 11).

**Table 1:** Vaccine composition, doses, route of administration and study groups.

Dose	I	II	III
Age of animal	24–48 h of life	14–16 days of life	28–30 days of life
Route of administration	Intranasally (IN)		Subcutaneously (SC)
The name of group (n)	**Experimental I (group CpG; n = 11)**		
Vaccine composition	40 µg rHsp60 *H. somni* + 80 µg rOMP40 *H. somni* + 10 µg CpG ODN2007 + PBS (add to 1000 µL)		20 µg rHsp60 *H. somni* + 40 µg rOMP40 *H. somni* + 10 µg CpG ODN2007 + PBS (add to1000 µL)
The name of group (n)	**Experimental II (group MPLA; n = 11)**		
Vaccine composition	40 µg rHsp60 *H. somni* + 80 µg rOMP40 *H. somni* + 75 µg MPLA + PBS (add to 1000 µL)		20 µg rHsp60 *H. somni* + 40 µg rOMP40 *H. somni* + 75 µg MPLA + PBS (add to1000 µL)
The name of group (n)	**Control (group Con; n = 11)**		
Injection (volume)	PBS (1000 µL)		
Dose volume per calf	1000 µl ; for 500 µl per nostril		1000 µl
Immunization schedule			

rHsp60 *H. somni*, recombinant heat shock protein from *Histophilus somni*; rOMP40, recombinant outer membrane protein from *Histophilus somni*; PBS, phosphate buffered saline; CpG ODN2007, synthetic oligonucleotides containing unmethylated CpG dinucleotides in particular sequence contexts; CpG, experimental group immunized with rOMP40 *H. somni*; rHsp60 *H. somni* and CpG ODNs; MPLA, monophosphoryl lipid A/experimental group immunized with rOMP40 *H. somni*, rHsp60 *H. somni* and MPLA; Con, control group.

The calves were enrolled in the study 24–48 h after birth. All calves were vaccinated three times, twice intranasally (IN) and once subcutaneously (SC). Intranasal vaccinations were performed between 24 and 48 h of life (sampling S1), and between 14 and 16 day of age (S2), and then (on day 28–30) subcutaneous immunization was performed (S3). At intranasal immunization one ml of vaccine was administered into two nostrils (0.5 mL each) with a syringe with an Intranasal Cannula intended for the administration of the Hipra vaccine in cattle (Hipra; Amer, Spain). Subcutaneous vaccination was performed using a needle (25G x 1) on the neck, and the final vaccine dose per animal was one ml.

During experiment blood samples, nasal secretions and saliva were taken five times:

**S1**- Immediately before the first intranasal immunization (24–48 h of life).

**S2**- Two weeks after the first immunization (14–16 days of age) and immediately before the second intranasal immunization.

**S3**- Two weeks after the second immunization (28–30 days of age), immediately before the third subcutaneous immunization.

**S4**- Two weeks after the third immunization (42–44 days of age).

**S5**- Two weeks after S4 (59–61 days of age).

Before each sampling, clinical examination of the calves was performed (rectal temperature ^°^C, nasal discharge, cough, heart rate, and breathing rate).

Blood samples were collected from the jugular vein using a needle (18Gx1 1/2″) and a 20 ml syringe (BD Becton Dickinson SSC; Franklin Lake, USA). Blood serum was harvested by centrifugation (10 min, 2500×g) within 2 h of collection. Serum samples were stored at − 80 °C. Nasal samples and saliva were collected with sterilized sponge (0.5 wide, 1.5 cm long) wrapped with linen string. The whole sponge was inserted into the nostrils, the entire sponge was tucked into the nasal passage and held with a finger so that the calf could not sneeze it out. The sponge was inserted into one nostril and mouth for approximately 30 s and then placed in 0.5 ml sterile phosphate buffered saline. Sponges were centrifuged at 23,227×*g* (Centrifuge MPW-380R, rotor ref 11770) for 20 min at 4 °C using insert from Salivette tubes (Sarstedt AG& Co. KG; Nümbrecht, Germany) and polypropylene falcon (15 mL; TPP Techno Plastic Products AG; Trasadingen, Switzerland). Nasal secretion and saliva samples were stored at − 80 °C.

Colostrum samples were collected from 19 dams, the colostrum from one dam was given to two calves from different experimental group, therefor the number of colostral samples in each group were: MPLA group, *n* = 6; CpG group, *n* = 8; Con group, *n*= 6). In eleven cases, the farm staff did not freeze colostral samples. Colostrum whey was prepared as previously described^48^.

### Determination of immunoglobulin concentration in nasal secretions, saliva, calves’ serum and dams’ colostrum

The concentrations of immunoglobulins (Ig) IgG_1_ and IgA in nasal secretion, IgA in saliva, and IgG_1_, IgG_2_, IgM, and IgA in calf sera and whey colostrum samples were determined using ELISA kits (Bovine IgG_1_/IgG_2_/IgM/IgA ELISA Quantitation Set Bethyl laboratories Inc.; Montgomery, USA). Different dilutions of the samples were used, and a supplementary file shows this in detail (see Supplementary Table S1).

The intra-assay CV for IgG_1_ and IgA concentrations in nasal secretions were 2.7% and 2.3%, respectively. The inter-assay CV for IgG_1_ and IgA in nasal secretions were 6.5% and 10.5%, respectively.

The intra-assay CV for IgA concentration in saliva was 1.6%. The inter-assay CV for IgA in saliva was 21.3%.

The intra-assay CV for IgG_1_, IgG_2_, IgM, and IgA in the sera were 2.1%, 1.7%, 2.2%, and 1.6%, respectively. The inter-assay CV for IgG_1_, IgG_2_, IgM, and IgA in the sera were 5.4%, 22%, 7.3%, and 9.2%, respectively.

The intra-assay CV for IgG_1_, IgG_2_, IgM, and IgA in the colostrum were 1.7%, 0.9%, 2.0%, and 1.7%, respectively. The inter-assay CV for colostrum samples was not calculated because colostrum samples were determined on one plate.

### Detection of anti-*H. somni* rOMP40 and anti-*H. somni* rHsp60 antibodies in nasal secretions and saliva by ELISA

Nasal secretions and saliva **(**sampling S1–S5) obtained from the experimental groups (CpG and MPLA) and the control group (Con) were examined for the presence of IgG_1_ (nasal samples only) and IgA antibodies against purified *H. somni* rHsp60 and rOMP40. Microplates (Nunc Maxisorp; Thermo Scientific; Waltham, USA) were coated with *H. somni* rHsp60 (3 µg/mL in 0.05 M carbonate buffer pH = 9.6, 100 µL per well) or with *H. somni* rOMP40 (3 µg/mL in PBS buffer pH = 7.4; 100 µL per well). The plates were blocked with PBS containing 1% Tween 20 (Merck KGaA, Darmstadt Germany; 200 µL/well; incubation for 90 min at 37 °C). Nasal secretion samples were diluted with PBS containing 0.05% Tween 20 (PBST) to a concentration of 30 µg IgA/mL and 10 µg IgG_1_/ mL for the detection of IgA and IgG_1_ antibody reactivity against *H. somni* rHsp60 and *H. somni* rOMP40 antigens, respectively. All saliva samples were diluted to 20 µg IgA/mL to detect IgA anti-rHsp60 and anti-rOMP40 antibody reactivities. When the concentration of IgG_1_ in nasal secretion samples was lower than 10 µg/mL (*n* = 15; five in Con group = 2xP1, P3, P4, P5; three in CpG group = P1, 2xP4; seven in MPLA = P1; 2xP2, P3, 2xP4, P5), the samples were not diluted. One hundred microliters of appropriate dilution/nasal secretion was added to each well, and the plates were incubated at room temperature (22 ± 2 °C) for 90 min on a rocker shaker (50 rpm). HRPO conjugates were diluted to 1:60000 for sheep anti-bovine IgG_1_ and 1:10000 for rabbit anti-bovine IgA (Bethyl laboratories Inc.). The plates were incubated at room temperature (22 ± 2 °C) in the dark for 60 min. The color reaction was developed using a supersensitive TMB substrate (100 µL per well; Merck) in the dark at room temperature for 15 min for IgA anti-rHsp60 and anti-rOMP40 antibodies, and 25 min for IgG_1_ antibodies. The reaction was stopped by 2 M H_2_SO_4_ (50 µl per well; P.P.H. Stanlab Sp.J.; Lublin, Poland). Optical density was measured at a wavelength of 450 nm using an ELISA Microplate Reader µQuantum™ (BioTek Instruments; Winooski, USA). All samples were assayed in duplicates.

The intra-assay CV for IgA and IgG_1_ reactivity against rHsp60 in nasal secretions was 2.6% and 2.8%, respectively, and the inter-assay CV was 11.7% and 4.0%, respectively. The intra-assay CV for IgA and IgG_1_ reactivity against rOMP40 in nasal secretions were 1.7% and 3.0%, respectively, and the inter-assay CV were 12.5% and 15.8%, respectively.

The intra-assay CV for IgA reactivity against rHsp60 in saliva was 1.9% and the inter-assay CV was 11.8%. The intra-assay CV for IgA reactivity against rOMP40 in saliva was 1.6% and the inter-assay CV was 11.8%.

### Detection of anti-*H. somni* rOMP40 and anti-*H. somni* rHsp60 antibodies in blood serum and colostrum by ELISA

Serum samples S1–S5 obtained from the experimental groups (CpG and MPLA), the control group (Con), and colostrum whey samples were examined for the presence of IgG_1,_ IgG_2_, IgM, and IgA antibodies against purified *H. somni* rHsp60 and *H. somni* rOMP40. Microplates (Nunc Maxisorp; Thermo Scientific) were coated with rHsp60 (3 µg/mL in 0.05 M carbonate buffer pH = 9.6, 100 µL per well) or with rOMP40 (3 µg/mL in PBS buffer pH = 7.4; 100 µL per well). The plates were blocked with PBS containing 1% Tween 20 (Merck; 200 µL/well; incubation for 90 min at 37 °C). Serum samples were diluted to 1:50 (to detect IgA antibodies against rOMP40 and rHsp60), 1:100 (to detect IgG_1_, IgG_2_ and IgM antibodies against rOMP40; to detect IgG_2_ and IgM antibodies against rHsp60) or 1:1000 (to detect IgG_1_ antibodies against *H. somni* rHsp60) with PBST. Colostral whey samples were diluted 1:100 (to detect IgG_2_, IgM, and IgA antibodies against rOMP40; to detect IgG_2_ and IgM antibodies against rHsp60), 1:500 (to detect IgA antibodies against rHsp60), 1:1000 (to detect IgG_1_ antibodies against rOMP40) or 1:8000 (to detect IgG_1_ antibodies against rHsp60). Then, 100 µl of the solution was added to each well and the plates were incubated at room temperature (22 ± 2 °C) for 90 min on a rocker shaker (50 rpm). The dilutions of HRP-conjugated antibodies were 1:60 000 for sheep anti-bovine IgG_1_; 1:20000 for sheep anti-bovine IgG_2_; 1:100000 for rabbit anti-bovine IgM, and 1:10000 for rabbit anti-bovine IgA (Bethyl laboratories Inc.). The plates were then incubated at room temperature (22 ± 2 °C) in the dark for 60 min. The color reaction was developed using a super-sensitive TMB substrate (100 µL per well; Merck) in the dark at room temperature for 15 min. The reaction was stopped by 2 M H_2_SO4 (50 µl per well; P.P.H. Stanlab Sp.J.). Optical density was measured at a wavelength of 450 nm using an ELISA Microplate Reader µQuantum™ (BioTek Instruments). All samples were analyzed in duplicate.

The intra-assay CV for IgG_1_, IgG_2_, and IgM reactivity against rOMP40 in the serum was 3.2% (on one plate, the reactivity of IgG_1_, IgG_2_, and IgM antibodies present in 15 samples was analyzed; 12 plates were compared), and for IgA was 4.1%, the inter-assay CV was 19.2% and 18.8%, respectively. The intra-assay CV for IgG_1_, IgG_2_, and IgM reactivity against rHsp60 in the serum was 2.2% (on one plate, the reactivity of IgG_1_, IgG_2_, and IgM antibodies present in 15 samples was analyzed; 12 plates were compared), and for IgA was 3.3%, the inter-assay CV was 18.1% and 20.1%, respectively.

The intra-assay CV for IgG_1_, IgG_2_, IgM, and IgA reactivity against rOMP40 in colostrum was 1.6%, 1.3%, 2.1%, and 1.3%, respectively. The intra-assay CV for IgG_1_, IgG_2_, IgM, and IgA reactivity against rHsp60 in colostrum was 1.5%, 1.6%, 1.4%, and 1.4%, respectively. The inter-assay distance was not calculated because the colostrum samples were determined on one plate.

### Acute-phase protein concentration in calves’ serum/plasma

The serum concentrations of haptoglobin (Hp) and serum amyloid A (SAA) were determined using ELISA kits (Cow Haptoglobin ELISA KIT Life Diagnostic Inc., West Chester UK; Multispecies SAA ELISA kit Tridelta Development Ltd., Country Kildare Ireland), and plasma fibrinogen (Fb) levels were measured as described by Millar et al.^49^.

The intra-assay CV for SAA was 5.2%, and the inter-assay CV for SAA was 14.3%. The intra-assay CV for Hp was 7.8% and the inter-assay CV for Hp was 3.2%.

### Statistical analysis

Statistical analyses were conducted using Statistica 13.1 (StatSoft Inc., Tulsa, OK, USA).

The experimental unit for each test was an animal. The data were checked for normality using the Lilliefors test. Only the hematocrit values showed a normal distribution. All other parameters showed a non-normal distribution; therefore, logarithmic transformation (log10) was performed. After transformation, only colostrum whey results (IgG_2_ OMP40, IgM OMP40, and IgG_2_ Hsp60) were normally distributed. All variables that had a normal distribution were tested using ANOVA, and post-hoc comparisons were performed using Tukey’s HSD test. Variables without normal distributions were analyzed using Kruskal-Wallis ANOVA with post-hoc multiple comparisons of mean ranks in cases where the three groups were compared.

The Wilcoxon matched-pair test was used to compare changes within each group separately. The Pearson correlation in IgA reactivity between nasal secretion and saliva was calculated.

## Results

### Determination of immunoglobulin concentration in nasal secretions and saliva

The concentration of IgA immunoglobulin in nasal secretion was in a range from 30.2 µg/ml to 3169.8 µg/mL (677.85 ± 607.4; 489.4 µg/mL mean ± SD and median, respectively), numerically the lowest concentration was observed in S1. The IgG_1_ concentration in nasal secretion varied from 5.9 to 116.39 µg/mL (36.9 ± 26.2; 28.5 µg/mL mean ± SD and median, respectively) and was not associated with sampling time. In saliva samples IgA concentration varied within the range from 18.2 to 1111.5 µg/mL (235.9 ± 298.4; 130.5 µg/mL mean ± SD and median, respectively). For more dilates, refer to the repository files.

### Detection of anti-*H. somni* rHsp60 and anti-*H. somni* rOMP40 antibodies in nasal secretion and saliva by ELISA

**Fig. 1:**
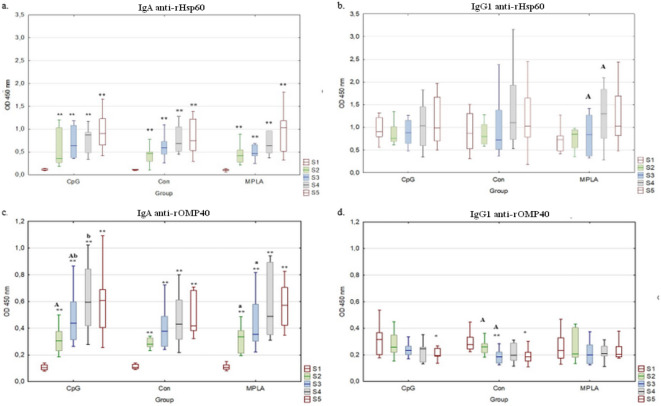
Determination of nasal secretion IgA and IgG_1_ antibody reactivity against *H. somni* rOMP40 and rHsp60. Box-plot graphs of IgA (**a**,**c**) and IgG_1_ (**b**,**d**) antibodies reactivity against *H.somni* rHsp60 (**a**,**b**), and *H.somni* rOMP40 (**c**,**d**) antigens in ELISA. Nasal secretions were obtained as follows: S1: before the first intranasal immunization (24–48 h of life); S2: two weeks after the first immunization (14–16 days of age); S3: two weeks after the second intranasal immunization (28–30 days of age); S4: two weeks after the third subcutaneous immunization (42–44 days of age); S5: two weeks after S4 (59–61 days of age). Individual study groups were labelled as CpG (vaccine formulation rHsp60 + rOMP40 + CpG), Con (control group), and MPLA (vaccine formulation rHsp60 + rOMP40 + MPLA). Significant differences within individual study groups between consecutive samplings (e.g., CpG S1-S2, S2-S3, S3-S4, and S4-S5) are labelled with the same uppercase letters (A) for *p* ≤ 0.01 or small letters (a, b) for *p* ≤ 0.05. Significant differences within individual study groups between S1 and other samplings (e.g., CpG S1-S2, S1-S3, S1-S4, and S1-S5) are labelled with two asterisks (**) for *p* ≤ 0.01, or an asterisk (*) for *p* ≤ 0.05. The median line across the box, lower, and upper boxes indicate the 25th percentile to the 75th percentile, and whisker non-outlier range.

The reactivity of anti-rHsp60 (Fig. 1a,b) and anti-rOMP40 (Fig. 1c,d) antibodies in both IgA (Fig. 1a,c) and IgG_1_ (Fig. 1b,d) classes present in nasal secretions did not differ significantly between the groups in subsequent sampling. In each group, the reactivity of IgA antibodies against *H. somni* rHsp60 (Fig. 1a) and rOMP40 (Fig. 1c) in S1 was significantly lower than that in the other samplings (S2-S5; *p* ≤ 0.01). In the case of IgA reactivity with *H. somni* rOMP40 in the CpG group (Fig. 1c), the intensity of the reaction in S2 was significantly lower than that in S3 (*p* ≤ 0.01) and the reactivity in S3 was significantly lower than that in S4 (*p* ≤ 0.05). The S2 reactivity of MPLA was significantly lower than that of S3 (*p* ≤ 0.05). In the case of IgG_1_ antibody reactivity with *H. somni* rHsp60 in the MPLA group (Fig. 1b), the reaction at sampling S3 was significantly lower than that at sampling S4 (*p* ≤ 0.01). Regarding IgG_1_ reactivity with *H. somni* rOMP40 in the CpG and Con groups (Fig. 1d), the reactivity of S1 was significantly higher than that of S5 (*p* ≤ 0.05). In the Con group, S1 and S2 were significantly higher than S3 (*p* ≤ 0.01; *p* ≤ 0.05, respectively).

IgA antibodies against *H.somni* rHsp60 and rOMP40 were also detected in saliva (see the repository files). Pearson correlation in IgA reactivity between nasal secretion and saliva was 0.74 for *H.somni* rHsp60 and 0.52 *H.somni* rOMP40 (*p* ≤ 0.05).

### Detection of serum anti-*H. somni* rHsp60 and anti-*H. somni* rOMP40 antibodies by ELISA

**Fig. 2:**
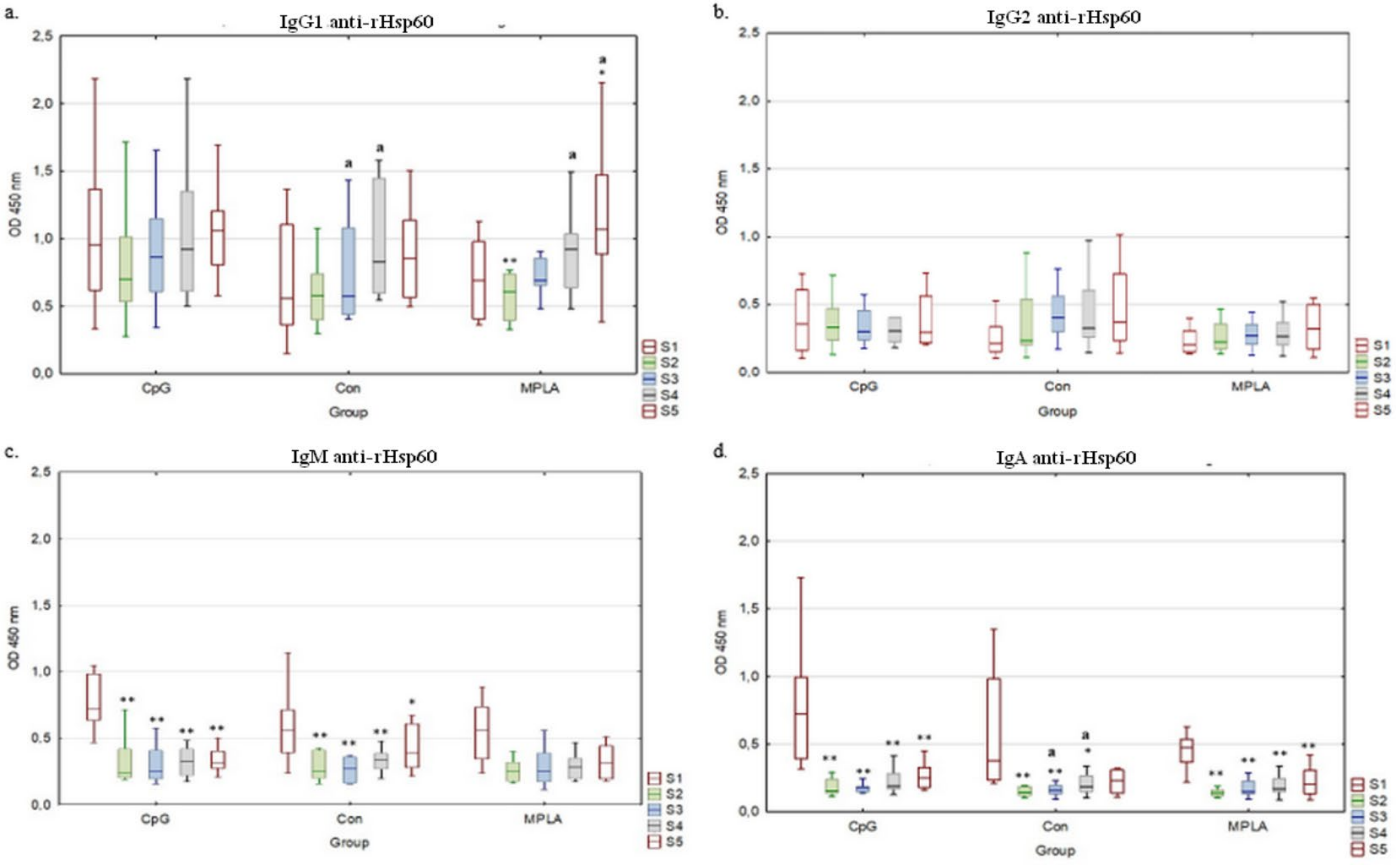
Determination of serum IgG_1_, IgG_2_, IgM, and IgA antibody reactivity against *H. somni* rHsp60. Box-plot graphs of serum IgG_1_ (**a**), IgG_2_ (**b**), IgM (**c**) and IgA (**d**) antibodies reactivity against *H. somni* rHsp60 antigen in ELISA. Serum samples were obtained as follows: S1: before the first intranasal immunization (24–48 h of life); S2: two weeks after the first immunization (14–16 days of age); S3: two weeks after the second intranasal immunization (28–30 days of age); S4: two weeks after the third subcutaneous immunization (42–44 days of age); S5: two weeks after S4 (59–61 days of age). Individual study groups were labelled as CpG (vaccine formulation rHsp60 + rOMP40 + CpG), Con (control group), and MPLA (vaccine formulation rHsp60 + rOMP40 + MPLA). Significant differences within individual study groups between consecutive samplings (e.g., CpG S1-S2, S2-S3, S3-S4, S4-S5, etc.) are labelled with the same lowercase letters (a) for *p* ≤ 0.05. Significant differences within individual study groups between S1 and other samplings (e.g., CpG S1-S2, S1-S3, S1-S4, and S1-S5) are labelled with two asterisks (**) for *p* ≤ 0.01, or an asterisk (*) for *p* ≤ 0.05. The serum dilution was 1:50 for IgA, 1:100 for IgG_2_ and IgM; 1:1000 for IgG_1_. The median line across the box, lower, and upper boxes indicate the 25th percentile to the 75th percentile, and whisker non-outlier range.

Anti-*H. somni* rHsp60 antibodies were detected in all examined sera from the control and experimental groups (Fig. 2a–d). The reactivity of serum anti-rHsp60 antibodies in all tested classes and subclasses did not differ significantly between groups in subsequent sampling. However, the reactivity of serum IgG_1_ antibodies in the MPLA group (Fig. 2a) in S1 was significantly higher than that in S2 (*p* ≤ 0.01), and significantly lower than that in S5 (*p* ≤ 0.05). In this group the highest reactivity was detected in S5, which was significantly higher than that in S4 (*p* ≤ 0.05). In the Con group, S3 reactivity of serum IgG_1_ was significantly lower than that of S4 (*p* ≤ 0.05). No significant differences in IgG_2_ antibody reactivity were detected within the individual study groups (Fig. 2b). Reactivity of serum IgM antibodies for CpG and Con groups in S1 was significantly higher compared to all other samplings (Fig. 2c, in CpG *p* ≤ 0.01, in Con *p* ≤ 0.01, except in the last sampling where *p* ≤ 0.05). The reactivity of IgA serum antibodies in all groups in S1 was significantly higher than that in the other samples (Fig. 2d, *p* ≤ 0.01, except for S4, where *p* ≤ 0.05, and S5, where no difference was noted in the Con group).

**Fig. 3:**
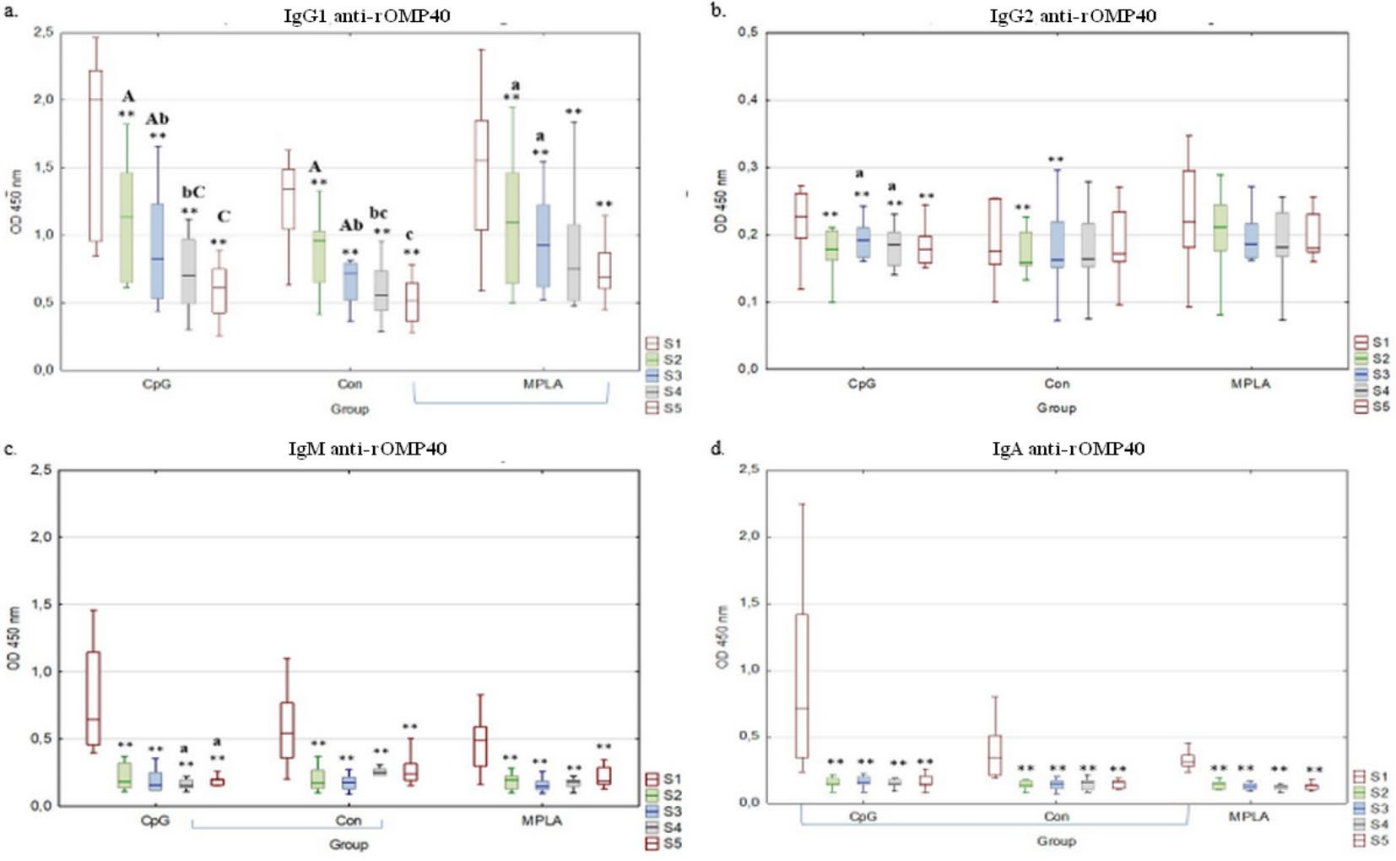
Determination of serum IgG_1_, IgG_2_, IgM and IgA antibody reactivity against *H. somni* rOMP40 antigen. Box-plot graphs of serum IgG_1_ (**a**), IgG_2_ (**b**), IgM (**c**) and IgA (**d**) antibodies reactivity against *H. somni* rOMP40 antigen in ELISA. Serum samples were obtained as follows: S1: before the first intranasal immunization (24–48 h of life); S2: two weeks after the first immunization (14–16 days of age); S3: two weeks after the second intranasal immunization (28–30 days of age); S4: two weeks after the third subcutaneous immunization (42–44 days of age); S5: two weeks after S4 (59–61 days of age). Individual study groups were labelled as CpG (vaccine formulation rHsp60 + rOMP40 + CpG), Con (control group), and MPLA (vaccine formulation rHsp60 + rOMP40 + MPLA). Significant differences within individual study groups between consecutive samplings (e.g., CpG S1-S2, S2-S3, S3-S4, and S4-S5) are labelled with the same uppercase letters (A-C) for *p* ≤ 0.01, or small letters (a-c) for *p* ≤ 0.05. Significant differences within individual study groups between S1 and other samplings (e.g., CpG S1-S2, S1-S3, S1-S4, and S1-S5) are labelled with two asterisks (**) for *p* ≤ 0.01, or an asterisk (*) for *p* ≤ 0.05. Significant differences between the study groups in the respective samples are labelled with brackets. Serum dilutions were 1:50 for IgA and 1:100 for IgG_1_, IgG_2_ and IgM. The median line across the box, lower, and upper boxes indicate the 25th percentile to the 75th percentile, and whisker non-outlier range.

Anti-*H.somni* rOMP40 antibodies were detected in all examined sera from the control and experimental groups (Fig. 3a–d). In S5, IgG_1_ antibody reactivity was significantly higher in the MPLA group than in the Con group (*p* ≤ 0.05; Fig. 3a). Serum IgM antibody reactivity in S4 was significantly higher in the Con compared to CpG group (*p* ≤ 0.05 Fig. 3c). Serum IgA antibody reactivity at S1 was significantly higher in the CpG group than in the MPLA group (*p* ≤ 0.05; Fig. 3d).

In S1, the serum IgG_1_, IgM, and IgA antibody reactivity against *H.somni*-rOMP40 antigen in all groups was significantly higher than that in the other samplings (S2-S5; *p* ≤ 0.01, Fig. 3a,c,d). In the case of the IgG_1_ antibody in the CpG and Con groups, a significant decrease in reactivity was observed between all subsequent samplings (S2-S3 *p* ≤ 0.01; S3-S4 *p* ≤ 0.05; S4-S5 *p* ≤ 0.01 in CpG and *p* ≤ 0.05 in Con group; Fig. 3a), whereas in MPLA, a significant decrease in activity was detected only between S2 and S3 (*p* ≤ 0.05). The reactivity of IgG_2_ antibodies in CpG in S1 was significantly higher than that in the other sampling (*p* ≤ 0.01). In this group, the S3 reactivity was significantly higher compared to S4 (*p* ≤ 0.05; Fig. 3b). The reactivity of the IgG_2_ antibody in the Con group in S1 was significantly higher than in S2 and S3 (*p* ≤ 0.01).

### Immunoglobulin concentration in calves’ serum

Immunoglobulin concentrations are shown in Table 2.

**Table 2:**
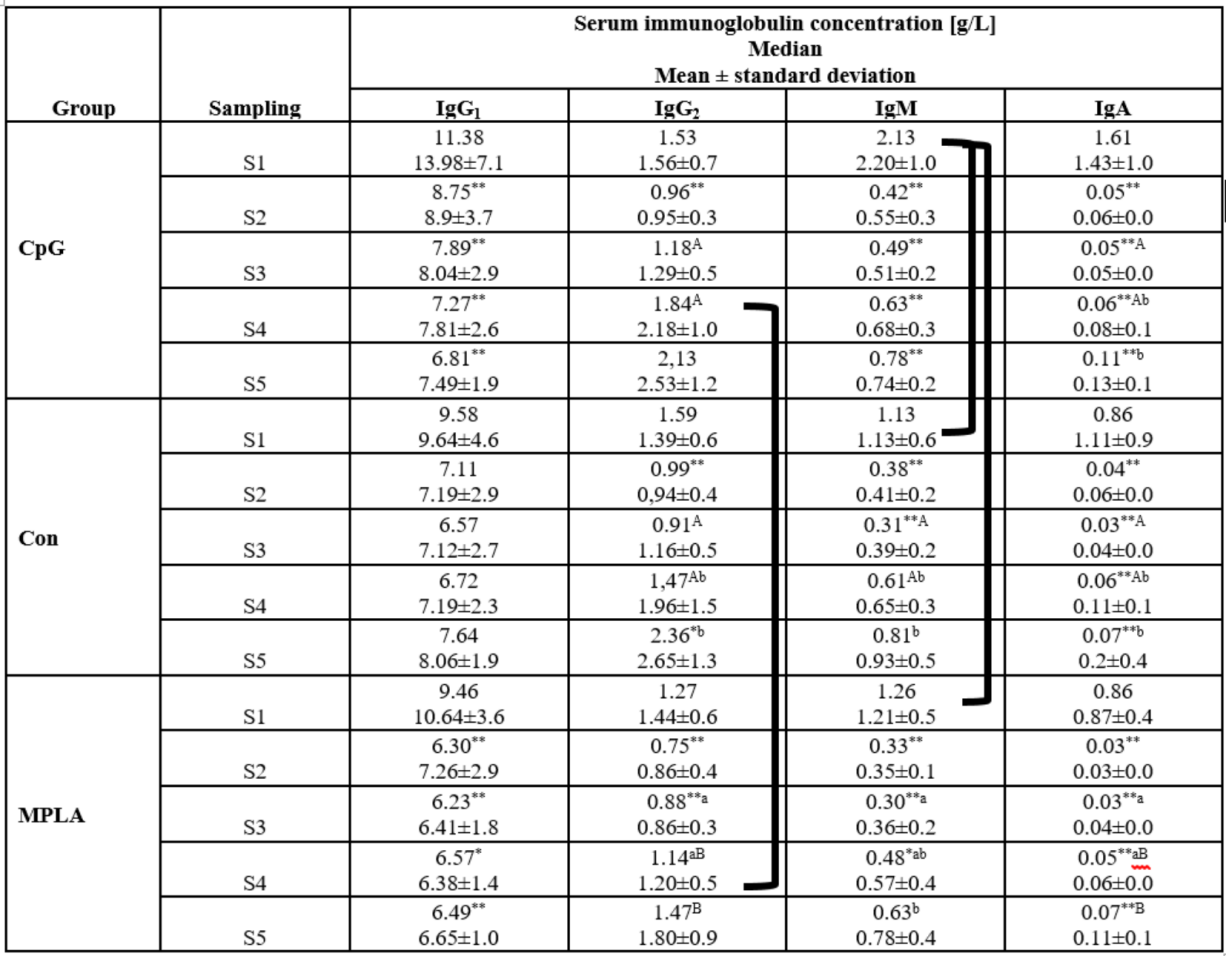
Median of immunoglobulin (IgG_1_/IgG_2_/IgM/IgA) concentrations in the serum of immunized calves.

Sera were obtained as follows: S1: before the first intranasal immunization (24–48 h of life); S2: two weeks after the first immunization (14–16 days of age); S3: two weeks after the second intranasal immunization (28–30 days of age); S4: two weeks after the third subcutaneous immunization (42–44 days of age); S5: two weeks after S4 (59–61 days of age). Significant differences within individual study groups between consecutive samplings (e.g., CpG S1-S2, S2-S3, S3-S4, and S4-S5) are labelled with the same uppercase letters (A-B) for *p* ≤ 0.01, or small letters (a-b) for *p* ≤ 0.05. Significant differences within individual study groups between S1 and other samplings (e.g., CpG S1-S2, S1-S3, S1-S4, and S1-S5) are labelled with two asterisks (**) for *p* ≤ 0.01, or an asterisk (*) for *p* ≤ 0.05. Significant differences between the study groups in the respective samples are shown in black brackets (*p* ≤ 0.05).

CpG, Con, MPLA, individual study groups; S1-S5, sampling.

In S1 serum IgM concentration was significantly higher in the CpG group compared to Con (*p* ≤ 0.05) and MPLA group (*p* ≤ 0.05). In S4, serum IgG_2_ concentration was significantly higher in the CpG than in the MPLA group (*p* ≤ 0.05). In both experimental groups (CpG and MPLA), the IgG_1_ concentration at S1 was significantly higher compared with other samplings (S2-S5; *p* ≤ 0.01; except of S4 in MPLA group, where *p* ≤ 0.05). The serum IgG_2_ concentration in S1 was significantly higher compared with S2 in all groups and in S3 in the MPLA groups (*p* ≤ 0.01). IgG_2_ concentration increased significantly between sampling S3-S4 (*p* ≤ 0.01 in CpG, Con; *p* ≤ 0.05 in MPLA) and S4-S5 (*p* ≤ 0.05 in Con; *p* ≤ 0.01 in MPLA). The serum IgM concentration was significantly higher in S1 compared with S2-S3 (*p* ≤ 0.01) in all groups. In the Con and MPLA groups, serum IgM concentration increased significantly in the following samplings from S3 to S5 (S3-S4 *p* ≤ 0.01 in Con and *p* ≤ 0.05 in MPLA; S4-S5 *p* ≤ 0.05). In all groups, serum IgA concentration in S1 was significantly higher than that in other samplings (S2-S5; *p* ≤ 0.01). Changes were also observed between samplings; IgA concentration increased significantly between sampling S3-S4 (*p* ≤ 0.01 in CpG, Con; and *p* ≤ 0.05 in MPLA) and S4-S5 (*p* ≤ 0.05 in CpG, Con; *p* ≤ 0.01 in MPLA). The highest sum of immunoglobulins in S1 was observed in the CpG group; however, it was not significantly higher than that in the other groups. The immunoglobulin concentration in S1 was significantly higher than that in other samples in the experimental groups; in Con, it was higher compared only with S2 and S3 (*p* ≤ 0.01) however S4 was significantly lower than in S5 (*p* ≤ 0.05).

### Concentration of acute phase proteins in calves’ serum/plasma

The concentrations of acute phase proteins such as serum amyloid A, haptoglobin, and fibrinogen are shown in Table S2 (see Supplementary Table S2).

There were no differences between the groups in the case of the investigated proteins. The highest SAA concentration was observed in S1. In MPLA group, significantly lower SAA concentration than in S1 was observed in S2, S4 (*p* ≤ 0.01) and S3, S5 (*p* ≤ 0.05). In CpG group, significantly lower SAA concentration than in S1 was observed in S3, S5 (*p* ≤ 0.05) and S4 (*p* ≤ 0.01). In CpG group, the serum SAA concentration was significantly higher in S3 than in S4 (*p* ≤ 0.05). In Con group significantly lower SAA concentration than in S1 was observed in S3, S4 and S5 (*p* ≤ 0.01). No significant changes in Hp and plasma Fb concentrations were observed in subsequent sampling.

### Immunoglobulin concentration and anti-*H. somni* rOMP40 and anti-*H. somni* rHsp60 antibodies reactivity in colostrum whey

The colostrum whey immunoglobulin concentrations are presented in Table 3.

**Table 3:**
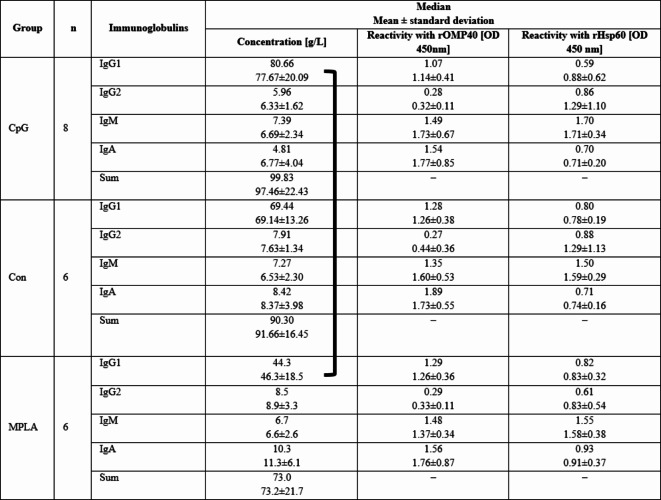
Median of immunoglobulin (IgG_1_/IgG/IgM/IgA) concentration and anti- *H. somni* rHsp60/rOMP40 antibody reactivity in colostrum whey.

CpG Con, MPLA, individual study groups; *n* amount of colostrum in individual study groups.

Significant differences between the study groups in the respective samples are labelled with black brackets (*p* ≤ 0.05).

The highest numerical immunoglobulin concentration was observed in colostrum whey obtained from cows in which calves were assigned to the CpG group (median = 99.83 mg/mL); however, there was no significant difference in the sum of immunoglobulins between groups. The difference was observed only in the IgG_1_ concentration, which was significantly higher in CpG than in MPLA (*p* ≤ 0.05).

The reactivity of anti-*H. somni* rHsp60 and rOMP40 antibodies in the IgG_1_ and IgG_2_ subclasses, and IgM and IgA classes in colostrum whey are shown in Table 3. High antibody reactivity with *H. somni* rHsp60 and rOMP40 antigens was observed in IgG_1_, IgM, and IgA classes. The highest reactivity with both antigens was observed with IgG_1_ antibodies (whey dilution 1:8000 and 1:1000, respectively). The lowest reactivity was observed for the IgG_2_ subclass (dilution 1:100). Antibody reactivity was similar in all the groups, and no significant differences were observed.

## Discussion

The nasopharynx can serve as a reservoir for BRD pathogens, which cause high morbidity and mortality in young cattle. Because early parenteral vaccination of calves is insufficient to protect them through a full period of susceptibility^50^, searching for a new intranasal immunization formula seems to be necessary. This study aimed to evaluate the early humoral immune response against selected, highly homologous, recombinant bacterial antigens (*H. somni *Hsp60 and OMP40) mixed with Pattern Recognition Receptor (PRR) ligands deposited intranasally twice, followed by a single subcutaneous vaccination in dairy calves during the first 60 days of life. CpG and MPLA were chosen as the adjuvants. CpG was formerly used as an adjuvant for mucosal immunization in calves^41^and has been successfully used for SC vaccination^36^. MPLA has also been used as an immunostimulator in vaccine formulations for SC^38^ immunization; however, to the best of our knowledge, calves have not been immunized IN with this adjuvant.

The new approach presented in this study was not only an attempt to induce an immune response on the mucosal surface after early IN immunization of calves (24–48 h of life), but also used selected *H. somni* recombinant proteins for this process. To date, *H. somni* rOMP40 and rHsp60 have not been used for IN immunization. The doses selected for intranasal immunization were 80 µg rOMP40 and 40 µg rHsp60 mixed with 10 µg CpG ODN2007 (CpG group) or 75 µg MPLA (MPLA group). At the third vaccination (SC), the concentration of *H. somni *recombinant proteins was reduced by half. Our previous studies showed that SC immunization of calves (two doses, age > 7 weeks)^25^and cows (five doses, age > 5 years)^51^ using 20 µg of *H. somni* rOMP40 emulsified with Emulsigen (adjuvant) induced strong immune responses in the IgG_1_ and IgG_2_ subclasses of antibodies. *H. somni* Hsp60 is a protein that is more immunogenic than the *H. somni* rOMP40. At SC vaccination, a dose of 10 µg of *H. somni *Hsp60 per animal mixed with oil adjuvant (Emulsigen) was sufficient to induce an intensive humoral immune response in calves (two doses, age > 4 weeks)^52^and cows (five doses, age > 5 years)^51^. As a new type of adjuvant was used in these trials, the amount of antigen for SC immunisation was doubled. The selection of protein and adjuvant concentrations for IN immunization was based on literature data. In a study by Muangthai et al.^41^, dosages of 50–100 µg of recombinant antigen (rOMP *P. multocida*) mixed with 10 µg of CpG ODN 2007 and administered IN to calves, induced a significant increase in sIgA levels in nasal secretions. Mulongo et al.^37^ for calf SC immunization used four recombinant proteins from *Mycobacterium bovis* at doses of 100 µg each mixed with MPLA. Therefore, the formulation composition used in this study appears sufficient to induce local and systemic immune responses.

The experiments showed that two intranasal and subcutaneous immunizations using the described compositions did not induce a significant humoral response against recombinant proteins in IgA and IgG_1_ antibodies on the mucosal surface compared with control animals. No significant differences were observed between the immunized and control groups. In nasal secretions obtained from all groups, the reactivity of IgA antibodies (against *H. somni* rHsp60 and rOMP40) in S1 was significantly lower than that in the other sampling (S2-S5; *p*≤ 0.01). This appears to be related to the postnatal development of MALT, as lymphocyte infiltration of MALT increases during the first weeks of life^2^. As calves grow and become mingled, the risk of stimulation by environmental bacteria increases. Moreover, the tested proteins are widespread in nature^26^and show high homology with the sequences of other gram-negative bacteria^23,25^. The presence of specific IgA antibodies in nasal secretions (sampling S2-S5) observed across all groups (including the control) may be attributed to a humoral immune response to homologous antigens of gram-negative environmental bacteria rather than the effect of immunization. Interestingly, the reactivity of specific IgG_1_ antibodies in nasal secretions did not show any correlation in subsequent sampling in all tested groups. IgG_1_ antibodies detected in S1 were probably of maternal origin, although contrary to IgA, transport of IgG_1_immunoglobulin to mucosal membranes from colostrum is much lower^53^. Our conclusion is in line with the results of Ellis et al.^54^, who showed the secretion of passively acquired IgG_1_ anti-BRSV antibodies into the nasal mucosa in calves fed with seropositive colostrum at 24 h of age. An increase in mucosal responses within a given group between adjacent terms, with no significant difference observed between the experimental and control groups, suggests that environmental factors exert an immunizing effect on mucosal membranes in calves. Mucous membranes serve as the primary site of antigen contact and the subsequent local immune response; thus, it is plausible that calves recognize the antigens utilized in the study through exposure to gram-negative bacteria. It is possible that such phenomena occur in instances where the quantity of antibodies acquired through colostrum is insufficient to further inhibit the immune response.

There is no information in the literature regarding the possibility of using recombinant proteins as antigens mixed with CpG and MPLA for IN immunization of neonatal calves (24–48 h of age). In one study^41^, calves were IN immunized three times at three-week intervals using a recombinant protein mix with CpG. Immunization induced both serum IgG and secretory IgA antibody production at a level significantly higher than that in the control group; however, the vaccinated calves were much older (4–6 months of age)^41^. In contrast to the possibility of using CpG as an adjuvant^41^, there is no information on IN immunization of calves using MPLA as an adjuvant. MPLA has been successfully administered intranasally and orally to mice, and induces a strong humoral response against recombinant antigens (50–100 µg of free or liposomal *Streptococcus mutans*crude glucosyltransferase)^55^. The results obtained in this study showed that an MPLA dose of 75 µg per calf is insufficient to induce an immunological response on the mucosal surface in all tested animals. However, the early IN immunization of calves is possible using commercially available vaccines, such as BioBos Respi 2 and 3 (Bioveta), Rispoval IBR (Zoetis), Rispoval RS + PI3 IntraNasal (Zoetis), Inforce™ 3 (Zoetis), Nasym (Hipra), and Bovilis Nasalgen 3-PMH (Merck Animal Health). These preparations can be administered intranasally during the first two weeks of life (a minimum of 3 days)^2,18^. However, these live vaccines contain modified live viruses or avirulent whole-bacterial live cultures^18^, which induce a strong immune response.

In the context of early IN vaccination of calves, establishing a proper immunization window seems crucial for the success of the process. In particular, the influence of maternal antibodies on the induction of immunity (in response to vaccines administered IN) and the duration of immunity following a single perinatal IN administration remain unresolved and controversial^17^. There are reports on effective immunization of calves on the first day of life using commercially available vaccines (e.g., based on BRSV modified-live virus)^56^however, as in most studies, experimental animals are deprived of colostrum in order to be free of maternal antibodies against selected pathogens^56,57^. Ellis et al.^57^ stated that in the case of 3-day to 8-day-old BRSV-seropositive calves IN immunized with a combination vaccine (containing modified-live viruses, and avirulent live cultures of *P. multocida* and *M. hemolytica*) and then challenged, the observed reduction of pneumonic lesions compared to seronegative-calves could be the effect of the transfer and re-secretion of maternal IgG_1_to the respiratory mucosae. Maternal antibodies can inhibit naïve B cells to differentiate into plasma cells and/or memory B cells in a maternal antibody titer-dependent manner^58^or neutralize the vaccine virus on the mucosal surface^57^. This may be considered as one of the potential factors contributing to the inefficacy of the vaccination protocol employed in this study in eliciting an immune response in calves. However, the mechanism of IgG_1_immunoglobulin transport across intact mucosal surfaces in the NALT in clinically normal animals remains unknown^54^. Ruminants are born essentially devoid of mucosal plasma cells in the nose^59^, implying that nasal immunoglobulin is derived from plasma^54^. It is worth emphasized that in the next reported studies Ellis et al.^17^ revealed that IN immunization of newborn calves with commercial antiviral vaccine were able to prime clinically relevant immune responses in the presence of maternal antibodies. The present data confirm the complexity of the issue of the influence of maternal antibodies on calf immunization.

In the context of the results obtained in this study, immaturity of the newborn’s nasal-associated lymphoid tissue must also be considered. It was shown, that NALT develops after birth^3^, it’s structure and functions are established by an interaction with commensal microbiota in the nasal cavity^4^. There is little information in the literature on how NALT develops and evolves in calves. It is only known, that in fetal calves (at 8–9 months of gestation), IgG + and IgA + cells could not be detected in the palatine and pharyngeal tonsils (MALT structures), which play an important role in local mucosal immunity at the entrance of the alimentary and respiratory tract. IgG + and IgA + cells were found in the area of both tonsils of 20-day-old neonates^60^(time of the second immunization). However, as the authors stated^60^, most IgGs in both tonsils are of maternal origin. Only a few cytoplasmic IgG + cells produce IgG in situ. Calve-origin IgG and IgA mRNA expression cells were detected in the tonsils of 3-month-old animals^60^. In our opinion, the immature nature of the NALT may have a significant impact on the obtained results.

One of the reasons for the lack of a successful IN immunization could be insufficient stimulation connected with an interaction between antigens and the mucosal surface. It is possible that formulations containing proteins mixed with CpG or MPLA were cleared from the mucosal surface too early and removed with nasal secretion, made its uptake by microfold cells (M cells)^61^and dendritic cells^62^impossible. These cells interact with viruses, bacteria, and other components of the microbiome^10^. Antigens, such as rOMP40 and rHsp60, are proteins that, unlike viruses, do not interact directly with epithelial cells^63^. Thus, the addition of suitable adjuvants could be crucial for inducing an immune response in the NALT structure. The adjuvants used in this study (CpG and MPLA) interact with pattern recognition receptors; however, the expression of PRRs in calf URT has not been analyzed^2^. PCR analysis showed that the TLR 1–10 genes were expressed in tracheal epithelial cells, tracheal tissue, and lung tissue^64^. However, it is unknown which PRRs are expressed by the mucosal epithelium and resident immune cells in the mucosa and submucosa of URT in cattle. Osman et al.^2^ stated that determining whether there are significant regional and age-dependent differences in the expression of PRRs in bovine URT may inform the formulation and delivery of IN vaccines in young calves. Therefore, the use of different immunostimulators/adjuvants should be considered in the future. A good candidate could be nucleoprotein nanoparticles (montanide monanide)^65^, nanoparticles (polyanhydride)^13^, microparticles (poly(-lactide-co-glycolide) polymer)^66^and ISCOMs (immune stimulating complexes)^67^. If the main problem in inducing a response is neutralization by maternal antibodies, liposome-based adjuvants may be a promising solution. Liposomes are versatile delivery systems for antigens, and they can be carefully customized towards the desired immune profiles by combining them with immunostimulators and optimizing their composition, physicochemical properties, and antigen-loading mode^68^. A strategy involving encapsulation^68^of recombinant proteins could allow us to avoid the interaction of maternal antibodies with antigens, and the adjuvant mechanism of liposomes, which is characterized by their ability to interact with antigen-presenting cells (APCs)^69^, would enhance the exposure of antigen and immunostimulators to the APCs and induce a strong humoral response.

To assess the systemic immune response after SC immunization, the reactivity of IgG_1_, IgG_2_, IgA, and IgM antibodies in the serum samples was evaluated. The tendency of IgG_1_ and IgA antibody reactivity in serum differed from that observed in nasal secretions. In the case of IgG_1_/IgA anti-rOMP40 and IgA anti-rHsp60 antibodies, the reactivity in S1 (before the first IN vaccination) was significantly higher than that in later samples. Analogous observations were made for IgM antibodies. The high initial reactivity of serum antibodies with antigens observed in all calves was probably due to colostral immunity, as we showed that specific antibodies were present in the dam colostrum. Bovine colostrum contains neutralizing antibodies against enteric pathogens, such as *E. coli*, *Pseudomonas aeruginosa*, *Staphylococcus aureus*, *S. pyogenes*, and *Campylobacter jejuni*^70,71^. These pathogens express proteins that are homologous to Hsp60 and OMP40 *H. somni*. No significant increase in *H. somni* Hsp60 and OMP40 antibody reactivity (in all investigated classes) between sampling S3 (before SC immunization) and S4 (two weeks after the third vaccination) was observed in the sera of the experimental groups. A significant increase between S3-S4 was detected only in the control group (IgG_1_ against both antigens and IgA against rHsp60). However, the intensity of the antibody reaction at S4 was not significantly higher than that in the experimental groups.

A comparison of serum antibody reactivity with literature data requires consideration of many aspects, such as the age of the animals, adjuvant used, protein concentration, and immunization schedule. It has been shown that calf SC and IM immunization using a formulation consisting of recombinant protein^34,35^or inactivated virus^36^mixed with CpG ODN 2007 or MPLA as adjuvant can be sufficient. Ioannou et al.^34^ immunized 9-month-old calves (twice, SC) with 50 µg of bovine herpesvirus 1 glycoprotein D (tgD) formulated with 25 mg CpG ODN (CpG). Placebo animals were injected with PBS only. The vaccinated groups had significantly higher levels of neutralizing antibodies and anti-tgD antibodies (in both IgG_1_ and IgG_2_subclasses) than the placebo group eight days after booster immunization. However, the vaccinated calves were older than those in the present study, and the adjuvant concentration was very high (250 times higher than that in our experiment). It was shown^35^that maternal antibody interference could be abolished by a formulation in which CpG ODN 2007 was mixed with Emulsigen (oil adjuvant). This formulation effectively stimulated the immune system of young calves. In experiments by Hurk et al.^35^, 3- to 4-week-old calves were immunized three times (at 3, 7, and 19 weeks of age) with 50 µg of tgD protein, 30% Emulsigen (Em), and 1 mg of ODN 2007. PBS was administered as placebo (control group). After booster immunization, the experimental group had significantly higher IgG titers than the placebo group. The formulation almost completely protected calves from BHV-1 challenge. These observations are consistent with those obtained by other researchers^34,37^. These results suggest that smaller quantities of CpG ODN 2007 (as used in this study) may require the additional use of Emulsigen as an adjuvant for SC immunization and could enhance the immunological response in neonatal calves.

For a second adjuvant (MPLA), Kathaperumal et al.^38^ demonstrated intensive immune responses and the protective efficacy of four proteins (85 A, 85 B, 85 C, and SOD) from *Mycobacterium avium paratuberculosis* mixed with MPLA. Neonatal (5–10 days old) calves were immunized subcutaneously twice. The experimental animals showed significant increases in antibody responses at 3 and 7 weeks after primary vaccination. In contrast to the results of our study, prevaccination serum samples from all animals did not react with any of the antigens. It is possible that anti-Hsp60 and anti-OMP40 antibodies present in the serum prior to immunization may react to and neutralize vaccine antigens. As a result, no substantial difference in specific antibody reactivity was detected between the vaccinated and unvaccinated animals.

In this study, immunoglobulin concentrations in the serum (sampling S1-S5) and colostrum were estimated. High levels of colostral Igs in the serum of calves are crucial for their health and growth during the first weeks of life^72^. The initially higher IgM class concentrations in the calves in the CpG group were likely the result of better passive immunity protection, as the other classes also had numerically (but not significantly) higher Ig concentrations. The results of the collected colostrum samples showed that the CpG group had the highest concentration in the IgG_1_ class; however, the concentrations that calves acquire in serum after colostrum feeding are not only related to the quality but also to the time of administration and volume of colostrum fed^73^. The highest concentrations of IgG_1_, IgM, and IgA were observed in S1 (after colostrum feeding) and decreased after the first 2 weeks of life. In the case of IgG_2_, which occurs at high concentrations in the serum of adult bovines^74^and is crucial for protection against pyogenic infections^75^, its concentration increased in calf serum from 14 days of life to the end of the experiment (sampling S2-S5). The observed trends in the changes in Igs concentration (sampling S1-S5) correspond with the results obtained by other scientists^48,76^. Nineteen colostrum samples were collected during the experiments. The IgG immunoglobulin concentration in the case of sixteen colostral whey samples (six from Con, seven from CpG, and three from MPLA) exceeded 50 g/L, which allowed us to determine that the obtained colostrum was of appropriate quality^77^ (see the repository files). The concentrations of IgG_1_, IgG_2 _and IgM immunoglobulins in the first colostrum of calf dams were similar to those reported by Gebert et al.^48^ (46.7 ± 4.9 g/L for IgG1; 5.5 ± 0.8 g/L for IgG2; 9.6 ± 1.1 g/L for IgM). IgA concentrations in more cases (twelve colostrum samples) were higher than those reported by Stephan et al.^71^ (3.2–6.2 g/L).

To evaluate the health of the calves, acute phase serum protein concentrations were measured. Fibrinogen, SAA, and haptoglobin are positive acute phase proteins that are used as indicators of inflammatory and traumatic diseases and as markers of infection in cattle^78^. During the experiment, no differences in plasma Fb and Hp concentrations were observed between the subsequent sampling and groups. Fb concentrations were similar to those reported by Gebert et al.^48^ and Gruse et al.^76^. The normal plasma Fb concentration in healthy calves does not exceed 6.45 g/L^79^. In this study, an Fb concentration above 6.45 g/L was observed in only two calves (MPLA group); in one of them, a small amount of unilateral cloudy nasal discharge was observed (see the repository files). Haptoglobin levels are often below the detection limit in healthy calves^72^and increase during chronic inflammation^80^. In healthy cattle, Hp is not detected in approximately 50% of individuals, and in the remaining 50%, its serum level is < 0.1 g/L (˂100 µg/mL). A concentration above 100 µg/mL has been reported during infection^72^. In this study, increased Hp concentrations were found in the serum of eight calves (one in the CpG group, five in the MPLA group, and two in the Con group). Three of them had diarrhea, and nasal discharge was observed in another three individuals (see the repository files). The SAA concentration in S1 was significantly higher than those in the other samples. The high level of plasma SAA in calves during the first days of life is probably induced by the absorption of inflammatory mediators, such as pro-inflammatory cytokines, from colostrum. Mediators may have crossed the neonatal intestine and stimulated hepatic production of SAA^81^. The concentrations observed during the first four weeks of life corresponded with the results obtained by Tóthová et al.^82^, Gruse et al.^76^, and Gebert et al.^48^. Gånheim et al.^83^ proposed acceptable SAA values below 25.0 mg/l for healthy calves aged 9–18 weeks. In this study, such low concentrations were observed in only 25 samples (one in S2, six in S3, and nine in S4 and S5). In most cases, the SAA concentration decreased during the experiment, which was similar to the tendency observed in other studies^48^. Calves in which SAA levels increased in samples S4 and S5 had diarrhea, nasal discharge, cough, or had undergone disbudding (see the repository files). The statistical differences found within groups in the laboratory parameters studied were mostly in accordance between groups; therefore, we did not associate them with the potential effect of vaccination.

## Conclusions

Double intranasal and additional subcutaneous immunization of one or two-day old calves using a vaccination formula consisting of *H. somni* recombinant proteins and TLR ligands as adjuvants under field conditions appeared to be ineffective. Several factors, including the presence of colostrum-derived antibodies on the nasal mucous surface and in serum, insufficient stimulation of the mucosal surface by the formulation utilized, and immaturity of the newborn’s nasal-associated lymphoid tissue, could influence the inhibition of the specific immune response. Establishing a proper immunization window and adjuvants for nasal vaccines against bacterial pathogens causing BRD in calves remain to be determined.

## Electronic supplementary material

Below is the link to the electronic supplementary material.

Supplementary Material 1

## Data Availability

The datasets supporting the conclusion of this article are available in the “Local and systemic humoral immune responses to selected recombinant bacterial antigens (Histophilus somni rHsp60 and rOMP40) administered intranasally and subcutaneously to dairy calves” repository; DOI:10.57755/g0wd-w889; https://bazawiedzy.upwr.edu.pl/info/researchdata/UPWRc254d629f7b34a0b92b58181e33211dc/.

## Abbreviations

rOMP40 *H. somni*: recombinant outer membrane protein 40 kDa obtained from *Histophilus* *somni*
rHsp60 *H. somni*: recombinant heat shock protein 60 kDa obtained from *Histophilus* *somni*
BRD: Bovine respiratory disease
NP: nasopharynx
CpG ODNs: synthetic oligonucleotides containing unmethylated CpG dinucleotides in particular sequence contexts
CpG: experimental group immunized with rOMP40* H. somni*, rHsp60 *H. somni* and CpG ODNs
MPLA: Monophosphoryl lipid A/experimental group immunized with rOMP40 *H. somni*, rHsp60 *H. somni* and MPLA
Con: control group
IN: intranasal immunization
SC: subcutaneous immunization
