# Supplementary material for: Local and systemic humoral immune responses to Histophilus somni recombinant antigens administered intranasally and subcutaneously to dairy calves
Source: Sci Rep. 2024 Nov 11;14:27567. doi: 10.1038/s41598-024-78605-x (PMC11555116; doi:10.1038/s41598-024-78605-x)
Supplement: Supplementary file 1 — Supplementary Material 1 [file 41598_2024_78605_MOESM1_ESM.docx]

Supplementary Table S1. Sample dilution used to determine immunoglobulin concentration.

| **Samples** | **Sampling time** | **Immunoglobulin** | **Dilution** |
| --- | --- | --- | --- |
| **Nasal secretion** | S1- S5 | IgG_1_ | 1: 500 |
|  | S1 | IgA | 1: 500 |
|  | S2-S5 |  | 1:1 000; 1:3 000; 1:5 000* |
| **Saliva** | S1-S5 | IgA | 1: 750 |
| **Serum** | S1-S5 | IgG1 | 1: 50 000 |
|  |  | IgG2 | 1: 15 000 |
|  |  | IgM | 1: 5 000 |
|  |  | IgA | 1: 500 |
| **Whey colostrum** | - | IgG1 | 1: 100 000; 1:300 000* |
|  |  | IgG2 | 1:20 000; 1:50 000* |
|  |  | IgM | 1:20 000 |
|  |  | IgA | 1:20 000 |

Legend: S1-S5: sampling. Samples were obtained: S1: before the first intranasal immunization (24-48 hours of life); S2: two weeks after the first immunization (14-16 days of age); S3: two weeks after the second intranasal immunization (28-30 days of age); S4: two weeks after the third subcutaneous immunization (42-44 days of age); S5: two weeks after S4 (59-61 days of age); *dilution depends on samples- different dilutions were done for samples with immunoglobulin concentrations above the last point of the standard curve.

Supplementary Table S2. Median of acute phase proteins (SAA/Hp/Fb) concentration in serum/plasma of immunized calves.

| **Group** | **Sampling** | **Acute phase proteins concentration** | | |
| --- | --- | --- | --- | --- |
|  |  | **Median** | | |
|  |  | **Mean ± standard deviation** | | |
|  |  | **SAA [mg/L]** | **Hp [µg/mL]** | **Fb [g/L]** |
| CpG | S1 | 178.8  194.25±121.27 | 3.2  9.68±21.58 | 4.6  4.32±0.71 |
|  | S2 | 91.9  117.35±78.73 | 2.8  20.23±57.07 | 4.5  4.52±1.05 |
|  | S3 | 71.2^*a^  71.68±27.83 | 2.5  10.89±27.55 | 4.5  4.45±0.95 |
|  | S4 | 58.6^**a^  49.87±27.37 | 2.5  4.51±6.65 | 3.9  4.16±1.06 |
|  | S5 | 54.4^*^  63.81±40.38 | 3.1  3.26±1.10 | 5.0  4.61±1.18 |
| Con | S1 | 159.3  172.83±70.07 | 3.7  4.03±2.33 | 4.3  4.24±1.12 |
|  | S2 | 98.2  131.73±123.83 | 3.3  5.06±4.54 | 4.9  4.68±0.93 |
|  | S3 | 51.0^**^  71.68±66.54 | 2.8  43.32±122.36 | 4.5  4.32±1.18 |
|  | S4 | 39.0^**^  45.47±30.84 | 2.6  2.62±0.45 | 4.4  4.27±0.89 |
|  | S5 | 62.4^**^  62.70±49.06 | 3.2  20.25±48.94 | 4.9  4.84±1.29 |
| MPLA | S1 | 118.8  169.51±103.36 | 2.9  3.70±1.92 | 3.8  4.04±1.14 |
|  | S2 | 74.7^**^  91.7±76.17 | 3.0  13.53±33.57 | 4.7  4.53±1.13 |
|  | S3 | 55.9^*^  69.68±50.15 | 2.4  34.99±68.10 | 4.1  4.16±1.51 |
|  | S4 | 63.3^**^  93.00±94.67 | 3.9  46.05±102.32 | 3.6  3.88±1.22 |
|  | S5 | 30.8^*^  52.70±55.32 | 3.2  3.2±0.69 | 3.3  3.81±1.09 |

Legend: CpG, Con, MPLA: individual study groups; S1-S5: sampling. Blood samples were obtained as follows: S1: immediately before the first intranasal immunization (24-48 hours of life); S2: two weeks after the first immunization (14-16 days of age); S3: two weeks after the second intranasal immunization (28-30 days of age); S4: two weeks after the third subcutaneous immunization (42-44 days of age); S5: two weeks after S4 (59-61 days of age); SAA: serum amyloid A; Hp: haptoglobin; Fb: fibrinogen. Significant differences within individual study groups between S1 and other samplings (e.g., CpG S1-S2, S1-S3, S1-S4, S1-S5) are labelled with two asterisks (******) for p≤ 0.01, or an asterisk (*****) for p≤ 0.05.
